# On Spasm, Languor, Palsy, and Other Disorders, Termed Nervous, of the Muscular System

**Published:** 1843-10

**Authors:** 


### Art. XII.

*On Spasm, Languor, Palsy, and other Disorders, termed Nervous, of the Muscular System.* By James Arthur Wilson, m.d., Fellow of the College of Physicians, and Physician to St. George's Hospital.—*London,* 1843. Sm. 8vo, pp. 201.

We hold it incumbent on any man who puts himself forward as the advocate of a new Theory to show, in the first place, that it is *called for,* by pointing out conclusive objections to that which was previously in vogue, and by demonstrating (or at least attempting to demonstrate) the freedom of his own from similar difficulties. When we took up Dr. Wilson's book, therefore, and gained from a cursory glance into its contents that the author has a new explanation to propound of the disorders of which it treats, we naturally looked for such a contrast. Having been unsuccessful in finding it—and having gathered, from a subsequent careful perusal of the work, that Dr. Wilson has given so little consideration to the physiological doctrines at present in vogue as to be incapable of making it,—we shall endeavour to supply the deficiency.

The readers of this Review need not be informed that we have, on all occasions, advocated the doctrine that muscular fibre possesses the property of *contractility* on the application of a stimulus, as an independent endowment; the existence of this property being dependent upon the maintenance of its organic structure by the nutritive process, and, therefore, upon the fitness of the blood to afford the requisite supply of duly-assimilated materials; and the readiness with which it may be called into action being dependent on a due supply of oxygen, which is also conveyed by the blood, and (in vertebrata at least) especially by the red corpuscles. Hence the contractile property of muscle may be impaired by causes acting through the blood; an insufficient supply of well-assimilated fibrin will prevent the due nutrition of the tissue, and an imperfect arterialization of the fluid will deaden the contractility so long as it continues.

Moreover there can be no doubt that poisons acting through the blood may impair or even destroy the contractility of muscle, by their direct influence upon the tissue. This is the case, for example, with lead, which is found to be deposited in the substance of the muscles which it paralyses; it is the case also with nitrate of potass and other neutral salts, which paralyse the heart as soon as they find their way (in sufficient amount) into its muscular substance through the coronary vessels. Many other substances, there can be no doubt, have an equally direct influence upon the muscular tissue when carried by the circulation into its capillaries; and if Dr. Wilson had applied himself to determine the operation of these by well-devised and well-conducted experiments, he would have done good service to science. That there are substances also which increase the contractility of muscles, there can be as little doubt; but, so far as we at present know, these substances act either by improving their nutrition or by increasing their supply of oxygen. The vegetable tonics may effect the former by their influence on the digestive and assimilating processes; whilst the agency of iron seems to be specially exerted in increasing the number of red corpuscles, and thereby aiding in the introduction of oxygen into the system.

But that any substances, with whose operation we are familiar, have the power of suddenly exalting the contractility of muscular fibre, to such a degree that the most violent and general spasmodic actions may be produced without any exaltation of nervous power, we altogether deny; and it was incumbent on Dr. Wilson to have *proved* this, before making this assumption (for it is no more) the foundation of his whole pathological system. It is true that he asserts that strychnia operates in this manner, but he brings no further evidence of it than that it produces violent spasmodic actions when circulating in the blood. No attempt whatever is made to controvert the received doctrine that it operates by inducing a highly-increased excitability of the Nervous system, especially of the spinal cord; a doctrine which appears to us fully established by the facts to be hereafter mentioned of its producing well-marked tetanic effects when directly applied to the spinal cord alone after the circulation has ceased. In order to prove the direct operation of the exciting cause of tetanus or any other agent upon the muscles, it would be necessary to examine how far the contractility of these muscles is increased by the circulation of these substances in the blood, *after* the muscles have been completely separated from their nervous connexions; and to observe their influence upon the heart and muscular coat of the alimentary canal, whose ordinary movements are but little affected by the nerves. Neither of these experiments have been performed, so far as we can learn, by Dr. Wilson, yet, upon the basis of a small number of facts, every one of which (as we shall presently show) is equally well explained upon the received doctrines, he erects the new hypothesis that, in the greater number of cases at least, general spasmodic, as well as paralytic disorders, are to be attributed to the direct morbid influence of certain agents, circulating in the blood, upon the muscular tissue.

Let us now turn to the nervous system; and examine its agency upon the muscles, and the influence of external agents upon it. The contractility of muscular fibre may be called into action by stimuli applied directly to itself; this is seen in the heart and alimentary canal, and also in individual fasciculi of the voluntary muscles. But no stimulus that we know of can produce a regular combined contraction of the latter, except one that acts through their nerves. In the living system, then, we cannot doubt that every contraction of a voluntary* muscle is occasioned by a stimulus acting through its nerves; whether this stimulus originate in the will, in an instinctive or emotional impulse, or in a reflex action of the spinal cord. The operations of the nervous centres, by which these effects are produced, are far more immediately dependent upon the blood than are those of muscular fibre. We check the circulation in the brain, and instantaneous suspension of its functions is the result. Impaired states of nutrition have an obvious influence upon the energy of the nervous system; and agents conveyed by the blood to the nervous centres, manifest their influence in a still more remarkable degree. Who can doubt that alcohol increases the activity of the operations of the brain, and that opium diminishes it? And why should we

* We use this term for convenience, although not strictly correct; since many of the contractions of these muscles are involuntary.

entertain any greater doubt that strychnia increases the excitability of the spinal cord by its direct influence on the nervous tissue, so that causes which would be in general inoperative to produce reflex movements, call them into violent operation?

We assert, without hesitation, that every one of the facts adduced by Dr. Wilson, to prove that the state of the blood has an influence in producing spasmodic disorders, may be explained equally well upon the received doctrine, that the morbid influence is exerted upon the nervous system. Consequently, there seems to us to be no occasion to quit this doctrine for one which, however specious, will be found to be entirely deficient in solidity at its foundation.

Dr. Wilson's views may serve, however, the good purpose of directing the attention of practitioners to the importance of considering general spasmodic diseases, and some forms of paralytic disease, as constitutional in their nature; and as originating, not so much in a local cause, as in a generally impaired state of the nutritive system, or in the retention of some injurious matter in the blood. It seems to us peculiarly necessary to bear this in mind, when applying Dr. Hall's views to practice; lest we should be led to look too exclusively at the state of the spinal system of nerves as the seat of the disorder, when the original fault is in the blood. We may explain our meaning by reference to hydrophobia. It is generally admitted that, in this terrible disease, a poison is introduced into the blood, which, after a longer or shorter period of latency, acts like a ferment upon the whole mass, and alters its character. The blood thus depraved, exerts an injurious influence upon certain parts of the nervous centres, and increases their excitability to such an extent, that the slightest causes are sufficient to induce the most violent spasmodic actions. It is quite possible that in traumatic tetanus, the similar result may be traced to an absorption into the blood of some disordered secretion from the injured part; for the *general* excitability of the spinal system of nerves on both sides of the body, which manifests itself, when the disease is once fully established, seems to us to point to the conveyance of a poison by the blood, through the whole of the spinal cord. In idiopathic tetanus, the constitutional or *humoral* origin of the disordered condition of the nervous system appears still more evident. And in those extraordinary forms of hysteria, which simulate these and other types of spasmodic disease, it is quite possible that we are to look for a similar cause; possibly, in many instances, the retention in the blood of matter which ought to have been got rid of through the genito-urinary organs.

That the nervous system has received, both from physiologists and pathologists, an undue share of attention, we are quite ready to admit. So far from being itself the source of life, as was long believed, it is only one manifestation of that life,—a manifestation of which one vast class of living beings is entirely destitute. But still the animal physiologist justly regards it as the instrument of certain functions, which increase in their variety, their importance, and their influence upon the organic life, as we trace them from the lowest animal up to man. And the pathologist, without any disposition to exaggerate its influence, must recognize in its disordered states the *immediate* cause of a vast number of phenomena, which ultimately may be very justly referred to the morbid condition of the blood, which is at the same time the *pabulum* of its nutrition, and the *stimulus* to its action.

With these preliminary remarks, we proceed to consider some of Dr. Wilson's leading doctrines in more detail.

Dr. Wilson is of opinion that the influence of muscular action on the blood, and, through the blood, on the function of organs in general, has been much underrated. "It is still with the flesh," he observes, "as it long was with the blood. Both have been unduly neglected in modern pathology." In the work before us, Dr. Wilson proposes to remedy this comparative neglect of the muscular system. He asserts the importance of the latter, and insists that it is at least on a par with the nervous system, of which it is in a great degree independent. That, in short, the muscular system is much more under the influence of the blood than the nerve:

"From within as from without, from the air we breathe, from the food we swallow, as from the inmost recesses of the most distant structures, there is a channel by which, more directly than by its nerve, most of what influences the muscle is received into its fibre. It is by the continuous universal blood, which while it bathes the fibre touches the air and mixes with the food,—it is by the blood in mass and in current that the muscle maintains its great and constant relations with the external agencies of matter, as with the elementary texture of every organ in the body." (p. 4.)

Dr. Wilson avers, contrary to the established opinion of physiologists, that this action of the blood on the muscular system needs not the intervention of the nerve to explain it. The effects are direct upon the muscle, but (with some inconsistency) he adds, and therefore upon the nerve as being one of the parts of its entire organized structure. There is some truth in the following opinion:

"Neither in health nor in disease is the muscle to be regarded merely as the organ of motion, as ministering only to an occasional and mechanical function; but, collectively and in the mass, as the most extensive of living structures, as continually employing and employed upon a large proportion of the entire mass of the blood, thus elaborating the great common material of the body and preparing it for circulation elsewhere. And this, be it remembered, is true of the muscle at all times, whether in action or repose...... The muscles, indeed, in their constant function of nutrition are with respect to the blood as glands, ever busy in separating from it the materials of their own growth, and restoring it in an altered state to the general current of the circulation." (p. 7.)

We experienced no little disappointment in finding, on a further perusal of Dr. Wilson's volume, that no development of this truth was intended. The changes effected in the composition of the blood by muscular action are almost unknown; consequently, an inquiry into these changes, viewing the muscles in their ultimate operation on the blood as glands, would have been exceedingly interesting. It is rather the changes induced in the functions of the muscular system by the blood, in a morbid state, that Dr. Wilson proposes to consider. Spasm, tetanus, chorea, muscular pains, languor, and palsy, are thus considered consecutively. In addition, there are a few words on mesmerism and magnetic sleep, on baths and mineral waters, and on exercise. A "memoir of fatal convulsions with renal disease, read before the College of Physicians in 1833," and woodcuts descriptive of the hot-air bath used in St. George's Hospital conclude the volume.

We will select tetanus as an example of the mode in which our author developes his views :

"The great character of this disorder is one of active defiance to the will in organs synonymous with the habitual exercise of its power. The contractions of the muscular fibre are never stronger than in the tetanic spasm, when by volition they are most opposed. In seeking for the principle of this countervailing agency in the muscle, we are led by our former inquiries at once to the blood. The blood, it is known, is independent of the will. It has the power of arranging its own particles in its own way,—it contracts and expands by a function inherent and peculiar to itself. It stirs instantaneously and simultaneously throughout its entire mass, thus swaying by direct movement the muscle which in bulk it forms. By the operation of certain external forms of matter on the blood, tetanus can immediately and at any time be induced. Again, it follows as an occasional effect of disturbance in the general business of temperature and nutrition, of which the blood is the chief agent and sole material, and with which the will has no concern.

"By thus considering tetanus in its wide and constant relations with the blood, we cannot fail to know it in its true character of a great constitutional disorder. Because it begins and ends with spasm of the voluntary muscles, tetanus has hitherto been classed with affections of the 'nervous system;' an undue limitation of the disease, for which there is no sufficient warrant by the symptoms or in its general pathology. Like fever, it pervades the entire system; and is special in the flesh, being common in the blood. A fever in truth it is, spasmodic and remittent in its character," &c. (p. 72.)

From a consideration of the symptoms generally, Dr. Wilson finds proofs that the disease is a fever. "In familiar phrase," he says, "tetanus may be termed the muscle fever of pathology." The toxicological action of strychnia on the system, is adduced as a triumphant proof of the doctrine that the blood is the first recipient and principal agent of the material influence under which tetanic spasm is produced. Dr. Wilson further asserts that "there are no good reasons for believing that the spasm, and other symptoms occasionally observed to follow on slight local injury, are induced by mere effects of 'irritation' from the nerves of the injured part." (p. 77.)

If Dr. Wilson intends to advance in the preceding quotation that a morbid state of the blood is necessary to the production of tetanus as a predisposing cause, we are not prepared to differ with him in opinion. But if it be advanced (as we think it is) by our author, that there is a specific poison or *materies morbi* in tetanus, circulating with the blood, as there is in the exanthematic and contagious fevers, and, by being brought with the blood into contact with the muscular fibre, developing tetanus, we at once join issue with him. There is no proof whatever that even when a poison (as strychnia) is introduced into the circulation and convulsions follow, its primary action is on the muscular fibre; but there is proof that it acts on the nervous system. Flourens showed by experiment, some years ago, that strychnia acted directly on the cerebellum, and in a case of poisoning by nux vomica, Orfila and Ollivier found much serous effusion over the cerebellum, and its structure softened. And anatomy has repeatedly demonstrated morbid changes in the nerves and spinal cord in cases of traumatic tetanus, and traced the morbid changes upwards from the irritated nerve to the central axis. For examples of this kind we refer Dr. Wilson to the cases communicated in 1827 to the Royal Academy of Medicine, by Lepelletier. Dupuytren, Beclard, Descot, Swan, Curling, and Froriep, will also afford Dr. Wilson convincing proofs that the irritation of the nerve passes directly to the central axis, and from thence is carried to the muscles.

We might add other observations of a similar character, but pass them by to notice Stilling's recent experiments with strychnia. This physiologist removed the entire viscera, namely, the heart, lungs, stomach, &c. from a great number of frogs; laid bare the brain and spinal cord from behind; and dropped a few drops of a solution of acetate of strychnia upon the latter. In *every* frog so treated, general tetanic spasms came on in about five minutes, and were re-excited by a touch, &c., just as if the animal had been poisoned by strychnine in the usual way. In these experiments the agency of the circulation was entirely excluded, yet if a small portion only of the spinal cord was touched with the solution of strychnia, the spasms were universal.* These experiments constitute a complete answer to the following assertion of Dr. Wilson's. "By no irritation, by no torture, however ingeniously devised, of any nerve or of any number of nerves in the body, would it be possible to establish a complete tetanus in the muscular structure under a given period of time, varying from forty-eight hours to three weeks." (p. 78.)

To say that "the blood entire is sensitive as the individual nerve of external impression, instantaneously and simultaneously perceived through all its distributions," (p. 86,) and thus to attempt to explain the peculiar phenomena of tetanus, is to found its pathology on what is really and truly a baseless vision, and we should think exclusively the property of Dr. Wilson.

The above exhibits Dr. Wilson's pathological views: let us now look at his treatment. Chorea in its acute form affords, according to our author, another variety of the "muscle fever," of which tetanus is the type. How does Dr. Wilson treat this "fever?"

"A young gentleman, aged thirteen, was brought to my house on the morning of October 5th, 1840, with severe chorea affecting the voluntary muscles of both sides of the body; and reported of about ten days' duration. On September 26th, while at school, he became unusually silent; and was observed to be continually engaged in unbuttoning his waistcoat, with general restlessness of manner. For some weeks previous his temper, naturally quick, had been remarked as irritable. The boy's health had always been considered delicate. On October 10th, when he was again brought to me, the spasm of the limbs was much aggravated; and he was unable to articulate. On October 13th I was summoned to him at his father's home, and found him convulsed as by tetanus or hydrophobia. His face was frightfully distorted; his tongue, black and dry, was forcibly protruded. By the violence of the spasm he was thrown repeatedly from the couch, on which he found no sleep. He was continually rending his clothes, and howling as if in agony." (p. 112.)

This patient died on the nineteenth day, of what we should have entered in our case-book as cerebral meningitis, commencing probably

* Untersuchungen über die Functionen des Rückenmarks und der Nerven.—Leipzig, 1842, p. 40. In 1838, Van Deen made many similar experiments on frogs, but with this difference, that the solution of acetate of strichnine was placed in the mouth. The results were the same. So early, however, as April, 1809, Majendie showed, that if the spinal cord be cut across, and one of the cut surfaces be placed in contact with a small quantity of upas, those parts only became convulsed which receive nerves from the portion of the spinal cord so treated.

at the base of the brain. Be this as it may, we need not comment on the following account of the treatment:

"The treatment, in the first instance, was by brisk purgatives, with tonic alterative medicines, including the Fowler's solution of arsenic. Latterly, morphia was given in small and repeated doses." (p. 112.)

It is evident to us, that in treating his subject Dr. Wilson has not had it fairly before him in all its relations. He has not apparently even made himself acquainted with the best established and now generally known neurological theories of the day. He writes almost as a physician of 1743 would write if he could rise from his grave and grasp a pen. For example: the cramps in the lower extremities from which pregnant women suffer, are now supposed to be dependent upon direct irritation of the lumbar and sacral nerves. Dr. Wilson thinks this explanation unsatisfactory, and he mounts his muscular hobby-horse to controvert it:

"It should be remembered," he says, "that in the process of gestation the general health is involved, and that the muscle is thus brought under influences affecting it in its entire structure, from which, in their effect on the blood and the fibre, spasm may ensue with as much likelihood as from pressure by the enlarged uterus on the trunk of the distant nerve."

We are quite ready to agree with Dr. Wilson in his opinion that the pathology of the blood has been hitherto much neglected, and that important results may be expected to follow from the study of pathological chemistry; but surely nothing can result from such loose hypotheses as he adopts, unsupported by experiment, and most illogically and obscurely stated. How can we possibly believe that the passions excite sensations independently of the nerves, and altogether through the blood? "Why," asks Dr. Wilson, in his enthusiastic devotion to the blood, "why should we suppose in such cases an agency of the nerve, intermediate between the material affording the impression and its physical effects first observed in the blood? Is anything in the body nearer to external influences than the blood, spread out as it is in the lungs in direct contact with the air, and with all that the air contains? Can this be affirmed of the nervous structure in either of its great divisions; of the double symmetrical nerves, or of those forming the ganglionic system?" (p. 38.) If Dr. Wilson had only asked himself the question, whether the blood had five senses or not; and whether it could hear, or see, or smell, or taste, or feel, he would surely have never published to the world such notions as these.

The quotations already given show Dr. Wilson's style of writing to be curiously quaint and obscure: it sometimes offends in other ways against good taste. The following sentences are examples:

"There is no state of the 'nervous system,' in its structure or function as generally understood, with which spasm of the voluntary muscles, however violent, is not compatible. Cæsar, great Julius, rose from earth-bound fits again to command the world; and he, the foremost among living men, like Cæsar in these unconscious struggles, still triumphs over himself." (p. 45.)

"By the term muscle, therefore, in our proposed inquiry will always be understood the living flesh in its combination of many parts, with the blood predominant in all; and by muscular action we shall never mean less than a result of the triple agency of nerve and fibre with the blood." (p. 8.)

